# “He Won’t Pay”: The Entanglement of Financial Abuse and Negligence in Swedish Child Maintenance Processes

**DOI:** 10.1177/10778012231185538

**Published:** 2023-07-07

**Authors:** Stina Fernqvist, Marie Flinkfeldt, Helena Tegler

**Affiliations:** 1 8097 Department of Social Work, Uppsala University, Uppsala, Sweden

**Keywords:** financial abuse, child maintenance, parents, case officers, thematic analysis

## Abstract

Since 2016, separated parents in Sweden are expected to pay child maintenance directly to each other unless special reasons, such as intimate partner violence (IPV), can be invoked. Problems with maintenance payments, which may involve expressions of financial abuse, have become a common topic in interactions between parents and the Swedish Social Insurance Agency (SSIA) that handles these cases. This article examines 132 phone calls to the SSIA, and the results show that payment problems are typically framed as relating to inability or negligence and not as possible indications of abuse. This highlights the need for training and capacity building regarding IPV in the Swedish welfare state.

## Introduction

Meanings of financial transactions and responsibilities within families have been renegotiated throughout history, in legislation as well as informal norms related to gender, age, and kinship. The outcome of these processes is manifested in unequal access to financial resources among the men, women, and children who, within a heteronormative frame, are viewed as “family.” This uneven distribution of resources can be conceptualized in relation to gendered inequalities in terms of financial power and abuse (Fawole, 2008; Meier, 1997; Walby, 2002) which are sustained in several ways in the modern welfare state, in spite of progress in legislation, policy, and practice. As the number of divorces and separations have increased steadily during the past decades and traditional norms stipulating the father as the breadwinner are on the decline in many countries, more children predominantly live with one parent, which has induced more extensive regulations of child support. In contrast to welfare schemes organized between state and claimant, child maintenance involves three parties: the state, the resident parent, and the liable parent. This makes it particularly complex, and the set-up of maintenance schemes differs substantially between countries, due to underlying structures, legislation, and organization of welfare distribution (see, e.g., Hakovirta & Skinner 2021; Natalier, 2018).

For instance, a comparative analysis between Finland, Germany, Spain, the UK, and the US showed that child maintenance often contributes significantly to the income of lone mothers and that there is considerable variation in the level of child maintenance between and within countries (Hakovirta and Jokela, 2019). Another comparison between 13 countries, conducted by Hakovirta and Skinner (2021), showed that differences in child maintenance distribution did not necessarily correlate with legislation or administrative rules, which indicates that the handling of maintenance is complex. Hakovirta and Skinner (2021) also note that much remains to explore regarding the sharing of care responsibilities between parents in separated families and suggest that institutions within childcare systems “are the best places to investigate this phenomenon as they generally have to respond to separated parents’ changing family practices” (p. 328).

Swedish welfare institutions are particularly interesting to analyze in this context due to the country's reputation as a “family-friendly” welfare state, which aims for gender equality via various forms of welfare incentives (Egeberg Holmgren, 2011; Eriksson, 2011; Lindvert, 2002). This entails, among other things, a preference for shared custody postseparation, which is largely based on an assumption that parents are willing and able to collaborate with each other even when they live apart. Some scholars have also identified Sweden as a good example with respect to child maintenance policy because of its “guaranteed maintenance scheme” (see, e.g., Skinner and Davidson, 2009), which could be viewed as an expression of a family-friendly welfare system. Guaranteed maintenance means that if the parent liable for payment neglects to pay, the resident parent can claim child maintenance from a state agency, which then collects the money from the liable parent. This guarantee was substantially limited by the maintenance reform in 2016, which we explore further in this article.

There is also an assumption in Sweden that it is in the child's best interest to have contact with both parents and that the parents can cooperate postseparation (Blomqvist & Heimer, 2016; Kurki-Suonio, 2000; see also Kaganas & Day Sclater, 2004). However, policymakers’ strive toward parental collaboration may overshadow the reality of conflict, intimate partner violence (IPV), and gender inequality (Eriksson, 2003, 2011; Harris-Short, 2010). In the context of the Swedish welfare state, this has been referred to as “the Nordic paradox” (see Wemrell et al., 2021).

IPV is often defined as expressions of physical, psychological, emotional, verbal, sexual, or financial abuse between partners where the majority of victims worldwide are women (Sardinha et al., 2022; WHO, 2019). Previous research has highlighted how various forms of financial abuse, where financial resources are used to exert control in intimate relationships, restrict victims’ financial scope for actions (Postmus et al., 2012; Sanders, 2015; see also Walby, 2002). Financial abuse has been conceptualized as a distinct form of abuse, but often linked to other forms of IPV (Eriksson & Ulmestig, 2021; Sanders, 2015). There is currently no straightforward legal definition of financial abuse in Swedish legislation. The National Board of Health and Welfare, however, states that financial abuse can be expressed through “financial threats, limitation of mutual financial assets or by forcing someone to commit financial unlawfulness” (National Board of Health and Welfare, 2022).

Previous research has also observed that the welfare state, through family policies and incentives that emphasize a family or child friendly perspective, may in fact facilitate financial abuse tactics since the dynamics behind IPV, rooted mainly in gendered inequality, remain largely ignored (Cook, 2021; Elizabeth et al., 2012; Fernqvist & Sépulchre, 2021; see also Eriksson & Ulmestig, 2021). Transactions and interactions regarding child maintenance have been observed as a context for exercising financial abuse or actions that may lead to financial vulnerability for the other party (Natalier, 2018). In this article, we contribute to this body of research by exploring and analyzing how financial abuse is visible, enabled, and handled in child maintenance assessment processes in Sweden.

### Child Maintenance in Sweden

Sweden passed the Income Maintenance Act in 1938, which was the country's first law regarding the support of children of divorced or unwed mothers and required the state to guarantee provision for the child and recover the allowance from the father (Bergman and Hobson, 2002). Another influential development in the formation of Swedish family policy a few decades later was a discursive and legislative shift in the construction of fatherhood in the 1970s, which contributed to establishing joint custody as the preferred option for separated parents (see Schiratzki, 1997). Today, Swedish family policies and legislation generally promote shared parenting after separation, whereby parents are expected to share the legal responsibility for their children and to have contact with each other (Eriksson, 2011). This preference became the norm in 1998 when courts could order 50/50 alternating residence, even in cases where one parent objected to it, with support from the Parental Code. Blomqvist and Heimer (2016) have observed that, although Swedish policymakers discussed the pitfalls of imposing shared custody in this way, it is often still decided that contact with both parents after separation is in the child's best interest. If the child resides an equal amount of time with each parent, no maintenance transaction is required. If the child, however, lives more with one of the parents, i.e., the resident parent, the other parent is liable for paying maintenance in some form.

In principal, maintenance is administered in two forms. The default model is *maintenance allowance*, stipulated by the Children and Parents Code, whereby the liable parent pays an agreed sum based on their income and actual costs for the child's sustenance directly to the resident parent. The other option is *maintenance support*, stipulated by the Social Insurance Code, whereby the resident parent receives a standardized sum from the Swedish Social Insurance Agency (SSIA), which then bills the liable parent. The payments can be routinized so that the applicable sum is automatically withdrawn each month from the liable parent's bank account. During the past decades, 87% of the resident parents receiving maintenance support in Sweden have been women (The Swedish Social Insurance Inspectorate (ISF), 2019).

In recent years, Swedish legislation has been pushed to emphasize parental financial responsibilities postseparation, whereby presumptions about parental cooperation have become increasingly visible in policymaking (see Bill 2014/15:145; SOU 2011:51). As part of this trend, child maintenance regulations were reformed in 2016 with the aim to promote parental cooperation and reduce the number of maintenance support cases handled by the SSIA. The reformed regulations stipulate that if the liable parent has paid the SSIA for 6 consecutive months, the SSIA will cease to administer maintenance transactions and the parents must organize the cash transfers privately (Bill 2014/15:145). To avoid such an arrangement, resident parents may claim “special reasons” for continued maintenance support, such as previous or ongoing abuse, in which case the SSIA's continued mediation of maintenance may be justified. Such exemptions are, however, not permanent: at the time of the data collection for this study, the case would be reassessed every 6 months, which often meant that parents who claimed special reasons once again had to present arguments for continued support. A more recent amendment in 2022 has, however, extended the duration of these exemptions, so that the time period between decision and reassessment is a minimum of 6 months and a maximum of 4 years (SFS 2010:110, 18 Chap. 9 a §). Nevertheless, the reform has led to a destabilization of a maintenance scheme that has previously been regarded as “guaranteed.” It could also be added that in the beginning of the reform, there was a lot of uncertainty regarding if, or how, claims of abuse should be verified by the parent. Claimants were therefore often asked to provide some sort of documentation, such as police reports or records from the social services (Fernqvist, 2020).

As mentioned above, one of the main arguments in the preparatory works leading up to the reform (e.g., Bill 2014/15:145) was the assumption that it is in the best interest of the child that parents collaborate with each other postseparation. Aside from a general view of parental cooperation as “inherently good” for the child, private maintenance arrangements were also expected to lead to larger maintenance sums (ISF, 2019) since they, unlike the standardized maintenance support administered by the SSIA, are based on the liable parent's income and the actual costs for the child. This understanding and framing of what is in the best interest of the child was, however, criticized before the implementation of the reform by Swedish interest organizations, politicians, and legal experts who alerted to a risk of (continued) abuse when parents with a history of abuse were encouraged to manage child maintenance privately. For example, the political party Feminist Initiative as well as Swedish society of lawyers expressed concern about how resident parents (usually mothers) would be affected by the reform, arguing that the reform relied on an “unrealistic view of parents’ will and capacity to collaborate” (Advokatsamfundet 2014, p. 2, author's translation). Through this reform, and especially the special reasons exemption, the issue of IPV in general has become more visible in the handling of maintenance in Sweden (see also Authors). It has also highlighted how a welfare state incentive may become an arena for financial abuse, which has not been investigated in the context of the Swedish welfare state. More research on this issue is needed and this article is a contribution to this field.

### Postseparation Violence, Financial Abuse and Maintenance

Financial inequality has been shown to be a foundation for financial abuse, mainly in heterosexual relationships, with gendered implications due to women's vulnerability through greater financial dependency on their partner (Meier, 1997; Walby, 2002). This may continue long after the relationship has ended, with consequences for women's material and social wellbeing (Bruno, 2018; Eriksson & Ulmestig, 2021). Postseparation abuse refers to tactics used to maintain control over a former partner through, e.g., stalking, physical assault, and psychological violence (see Brownridge, 2006: Elizabeth 2017; James-Hanman & Holt, 2021). For example, a comprehensive Swedish survey showed that one-third of women who had experienced IPV in a relationship were also abused after the separation (Lundgren et al., 2001). This form of abuse can include features of financial violence, both directly by depriving the victim of money and/or material assets or by neglecting or sabotaging agreements regarding finances (see also Miller & Smolter, 2011). Financial abuse may also be exerted by, e.g., procrastinating or sabotaging legal and administrative processes associated with separation or divorce (Miller & Smolter, 2011; see also Diesen, 2016), or by withholding maintenance payments (Natalier, 2018). In addition, other forms of IPV may result in financial vulnerability also after a relationship has ended (see, e.g., Brush, 2003).

In general, financial abuse can be exerted through one partner's control of jointly owned financial resources and withholding information about the financial situation or activities but also through acts such as destroying the partner's belongings or depriving the partner of basic resources such as food, clothes, or medicine (see, e.g., Adams et al. 2008; Anitha, 2019; Postmus et al., 2012). The woman's financial agency may of course differ depending on the cultural context. Surveys have, for instance, showed that regions in sub-Saharan Africa has the highest percentage of husbands (around 65% in Nigeria and Malawi) making decisions regarding household finances, which means that many working women have little or no influence to decide how the households’ finances are spent (Fawole, 2008). Sanders (2015) argues that intersections between various forms of violence can be manifested in women's financial dependence, which may prevent them from leaving an abusive spouse. This is also visible in poor women's vulnerability to abuse on a general level and when “partners use a variety of intentional tactics that negatively affect women's economic well-being” (p. 4). In general, this could be perceived as a form of structural violence where individual experiences of abuse can be linked to the social structure or in social norms that in different ways may facilitate that abuse (see Galtung, 1969; Sinha et al., 2017).

When it comes to child maintenance, withheld payments and other tactics to obstruct maintenance arrangements can be a way to maintain financial, often gendered, dependency, and vulnerability long after the relationship has ended, with little or no interference from the welfare state. Some welfare schemes may even facilitate financial abuse by enabling perpetrators to sustain a financial advantage and undermine their ex-partners’, in practice often mothers’, financial autonomy (Cook, 2021; Natalier, 2018). Previous research has shown that the Swedish maintenance reform, due to the increased contact between separated parents, has enabled financial abuse in several ways (Fernqvist, 2020; Tegler et al., 2023). In addition to not paying the correct amount, or paying late, liable parents may use areas of contact attached to money transfers (e.g., bank statements) to verbally insult or threaten resident parents (Fernqvist & Sépulchre, 2021).

There are also indications that the 2016 maintenance reform has provided opportunities for expressing financial power and abuse through diligence and “good behavior” by the liable parent when handling maintenance transactions (Tegler et al., 2023). As stipulated by the reform during the time of this study, the SSIA will cease to intervene after 6 months if the liable parent pays orderly to the agency (a time span which has since changed from 6 to 12 months, see SFS 2010:110, 18 Chap. 9 a §). Managing payments to the SSIA could thus be a way to get renewed contact with the resident parent. It may also work as a strategy to give a good impression in custody proceedings due to the aforementioned significance of parental collaboration postseparation that informs Swedish family policy in general, with a particular emphasis on the importance of responsible and present fathers (Diesen, 2016; Bruno, 2018). The risks and discomfort that increased contacts related to maintenance payments (physical encounters, text messages, and financial transactions) may have, mainly for the resident parent, are likely to be downplayed in that context. The aim of this study is thus to explore the practical entanglement of payment problems, financial neglect, and financial abuse following the maintenance reform in 2016, and this article provides new insights on how institutional encounters regarding these matters are played out and how accounts of financial abuse are handled by a government agency.

## Methods

This study is a qualitative analysis of a large database of phone calls from parents to the SSIA customer service, where all calls relating to child maintenance (in total 649 calls; ∼55 h of audio data) have been examined. Other forms of communication between the SSIA and claimants (e.g., e-mails or chat messages) have not been a part of our data as the SSIA only uses such channels for general queries and encourages clients who raise case-specific concerns to instead call the telephone service. The calls were recorded in 2016 and 2017, i.e., at the time when the reform was launched and many parents received a letter informing them that the SSIA would no longer function as an intermediary party. The recorded customer service line is the default mode of contact for parents with questions or objections relating to the reform, and the recorded calls thus provide unique insight into the concerns that the reform raised at the time of its implementation. In addition, investigating naturally occurring interaction enables direct insight into what kinds of problems that are treated as institutionally relevant and how they are dealt with *in situ* (Tegler et al., 2023; Iversen et al. 2021).

Nine SSIA case officers working with maintenance support gave written informed consent to participating, and all their calls were subsequently recorded. Four of these are featured in the calls presented in this article, but the identified patterns were not specific to them. The case officers participating in the study function as first-line gatekeepers and can decide what should be documented in each case, how the parents should be addressed, or if the parent should be referred to a “back office” case handler for further investigation and assessment of special reasons warranting maintenance support.

Callers were given oral information about the study in a prerecorded message while waiting to be connected to a case officer and were encouraged to tell the case officer if they wished to be excluded. No additional personal information (beyond what is mentioned in the calls) was collected. The study has been approved by the Ethical Review Board in Uppsala, Sweden (reference no. 2016/073).

Data were analyzed stepwise. First, all recorded phone calls regarding maintenance were listened to, and notes were made about call purpose, descriptions of violence or conflict, etc. One hundred thirty-two calls (∼17 h), from unique callers, featured talk about conflict and/or violence before, during, or after separation, and these were selected for further analysis and transcribed in full. In order to detect the interplay between experiences of IPV and state welfare regulations and routines regarding child maintenance, it was important to also include calls where conflict of various kinds was brought up. A reason for this is that disclosing IPV is associated with a range of difficulties, so that abused victims (if raising the topic at all) may normalize or minimize their experiences (see Greatbatch & Dingwall, 1999). A more inclusive approach may therefore be necessary when studying naturalistic interactions. The callers in our sample comprised 87 women (75 resident mothers and 12 liable mothers) and 45 men (10 resident fathers and 35 liable fathers). For the purpose of this paper, we have focused on calls where mothers address problems with payments in some form. Both liable and resident parents addressed conflicts in relation to payments, but while resident mothers, in particular, described events consistent with IPV, liable fathers typically argued that they had to pay too high amounts or reported conflict regarding other aspects of the maintenance arrangements (e.g., not being able to see the child). Furthermore, the study is limited to parents that have lived in heterosexual relationships. The absence of other forms of parental constellations is not an intentional limitation but follows from the data, since all parents in our analyzed calls themselves made their relationships visible as heterosexual. While the results from this study do not rule out the possibility that similar patterns can be found for nonheterosexual parental constellations postseparation, exploring this is a task for future research.

After forming the subset of 132 calls, transcripts were read through repeatedly and coded in the software program NVivo, in accordance with thematic analytic (TA) methods which aim at identifying, analyzing, and reporting patterned meaning in qualitative data (Braun & Clarke, 2006, 2021; Vaismoradi et al., 2013). The coding in the study as a whole was conducted in line with what Braun and Clarke (2021) call a reflexive thematic analysis, which is an approach focused on developing codes into themes that are underpinned by a central organizing concept. Here, the main focus has been to explore and analyze how IPV are made visible in the calls, and the themes developed for the purpose of this article are centered around how financial matters are made relevant in this context. The examples used in the paper have been selected based on how well they illustrate patterns identified in the analyzed data as a whole. We have strived to maintain as much interactional detail as possible, but to improve readability and clarity, some edits have been made. The excerpts have been translated from Swedish to English and all names, geographical references, etc. have been changed throughout to preserve participants’ anonymity.

## Results

The maintenance reform has made it necessary for SSIA case officers to distinguish between abuse and conflict, since previous experiences of IPV—i.e., “special reasons”—may grant resident parents continued right to maintenance support (Tegler et al., 2023). However, our analysis in this paper shows that parents’ apprehensions regarding the risk for payment problems tend to be treated as a practical problem, rather than a possible indication of financial abuse or linked to abuse in any other form. Given the study's focus on first-line responding case officers, the discretionary space for asking questions of a more investigative character is limited. The case officers nevertheless need to elicit sufficient information to decide whether to transfer the case to back office investigators for further handling or dismiss the parent's claims directly, and they are therefore not explicitly instructed to avoid asking questions regarding possible abuse.

The analysis is structured around three themes that we found to be prominent in the data. In the first section, we show how financial abuse is described as a part, or consequence, of a broader picture of various forms of violence. The second section explores the difficulties of distinguishing financial negligence from acts of abuse in a social insurance context. Finally, case officers’ responses to accounts of abuse are discussed with a focus on how financial transactions, and the contact they entail, are framed as safe by the SSIA.

### “Money Problems” Linked to Various Forms of IPV in Maintenance Calls

Callers in our data rarely raise experiences of IPV in explicit terms (see Tegler et al., 2023 for a discussion), but when they do, their accounts tend to also involve descriptions about financial concerns and anticipation of difficulties reaching an agreement about maintenance payments with the other parent. In this section, we show how “money problems” of this kind can be part of a broader picture of IPV.

In the first example, the caller accounts for her request for continued maintenance support by describing lack of contact, a “tense situation,” and a previous threat of suicide:

Ex. 1 (call 1988)

Parent: Me and their father have not had any contact. I haven’t talked to him since April last year … so the situation is tense.

Case officer #1: Yes

P: How can I explain? It was a super difficult journey for me and the kids when the maintenance was settled and he threatened to kill himself when they were little.

CO: Yes

P: And his mom thought “well, he can just come by your place with some groceries now and then instead”. It was really hard. I just remember crying and feeling bad and it was hard, but it has worked since then.

CO: Yes

P: But now everything is torn up again (…) So I’m begging you, can it just continue the way it has?

Threating to commit suicide can be part of controlling behavior and IPV (see, e.g., Lundgren et al., 2001). In this example, the caller describes the problems with maintenance payments as part of a broader set of issues including this threat and makes explicit the consequences for her (“crying and feeling bad”). She also orients to financial vulnerability as linked to the dysfunctional relationship as a whole, singling out food as the type of thing the father's contribution might cover. Notably, the problems are claimed to reach beyond the relationship between the parents, also affecting the children, and the caller's plea for continued SSIA involvement can thus be heard to be not only for her sake but also for the children's.

In the next example, the caller similarly links problems with money to (other) forms of violence. The caller has requested that the maintenance should be managed by the SSIA, and the case officer has resisted this, informing the caller that “special reasons” are required in order to avoid direct payments. The caller then states her reasons—she has been threatened by her ex-partner, and the police are involved—which is related to financial arrangements:

Ex. 2 (call 1886)

P: Okay but then I have special reasons in that case because he has

threatened me before and I have reported him to the police and it has also

been in situations where money has been involved.

The next example illustrates specifically how maintenance can be manipulated to exert financial abuse. In this call, the mother describes in detail various acts of abuse after finishing a long relationship, concluding that “I can’t see that person again, then I wouldn’t know, we live on the sixth floor, what would happen then? Will he thro- toss me from the balcony next time? I might not survive then.” It then turns out the father has filed for maintenance support from the SSIA, claiming that the children live solely with him, and has then hidden all SSIA correspondence from the mother, so that she has worked up a large debt:

Ex. 3 (call 2340)

CO #2: Since we never got the letters in return we assumed that you received them.

P: Yes

CO: Therefore, a decision has been made and you have been notified and God knows what…

P: Yes

CO: So, like, you have debts piling up here…

P: Wow … Oh God, I am panicking, don’t know what to do

Beyond illustrating a way in which the maintenance scheme can be used strategically to exert abuse, this example shows how maintenance payments may be intimately linked to other forms of abuse. While there are no instances in our data where financial problems are explicitly *labeled* as abuse, the analysis in this section thus shows that such problems are part of how IPV is described in this context and our analysis also points to how previous experience of IPV is associated with financial vulnerability. Overall in our data, however, parents tend to not label their problems as IPV. While they describe issues that might be implicative of violence, only 11 callers state explicitly that they have been subjected to abuse. Meanwhile, parents—mostly mothers—typically claim that the other parent will not pay maintenance directly to them or express worry that moving from maintenance support to managing maintenance transactions themselves will not work. This theme will be explored further in the following section.

### “He Won’t Pay”: A Question of Abuse or Negligence?

Since the “special reasons” exemption includes financial abuse, case officers might be expected to explore indications of financial problems relating to maintenance payments, to assess whether they could be grounds for continued maintenance support via the SSIA. In our data, however, case officers tend to treat descriptions of financially related problems between the parents as practical issues rather than a possible indicator of IPV. In this section, we show how callers’ descriptions of payment issues can make it difficult to distinguish abuse from negligence and how case officers tend to treat such accounts as indications of the latter.

In the example below, the caller describes a situation where the father previously has not paid as agreed, and she therefore wishes for the maintenance payments to be administered by the SSIA:

Ex. 4a (call 2084)

P: Well my reason is that … well, it has been working on and off and he is not paying as he should. Plain and simple. He can call and say ‘I can’t pay now, can I do it later’, or just doesn’t transfer the money. So I want the SSIA to handle this.

While the father's nonpayment is not labeled as abuse in this call—and the mother quotes the father as claiming to be *unable* to pay—it cannot from the description given be ruled out that the behavior is intentional. It is also clear that it poses a problem for the mother and that the arrangement enables financial control in a way that affects her life. Rather than probing into the details of this situation to ascertain that it does not constitute IPV, however, the case officer informs the mother that payments mediated by the SSIA require explicit forms of abuse, thus treating the mother's problem as falling outside of this definition:

Ex. 4b (call 2084)

CO #3: What has been established is mainly that, like, if there are threats and violence, that may be a reason that might … that could make [the maintenance] remain at SSIA. That's all that has been said in the legislation.

P: Mm hm.

CO: Eh … And if there are no other reasons, like if one doesn’t think … it will not be managed…there is really no reason for the SSIA to (inaudible)

P: Although (in a louder voice) it has been like this for a long time?

The case officer here makes explicit that to anticipate that “it will not be managed” is not sufficient reason for the SSIA to intervene. By such means, the case officer discourages any further tellings about systematic financial abuse that the caller might have experienced and treats IPV and irregular payments as separate things. This institutional response thus ignores the subtle ways in which financial oppression and abuse may be exerted and how a position of power may enable “paper abuse” (cf. Miller & Smolter 2011) that in various ways forces the other parent to engage in administrative processes. The next example illustrates how having to deal with irregular payments from the liable parent may wear the resident parent down:

Ex. 5 (call 1886)

P: Our daughter turns eight this summer and it is ten years left until she turns eighteen, that is far too long time for things to go wrong if you know what I mean.

CO #2: Mm, but if we decide that he should pay directly to you and he doesn’t, there is still a possibility for you to receive maintenance via the SSIA. We are not saying that you should go without it, but rather urging the parents to…

P: I understand but there will be problems and it's like … it takes strength and energy and it will be a constant administrative task, I will have to make calls and e-mail him and, yeah…

While the case officer raises the possibility to reinstate the maintenance support if the mother would not receive the money from the father directly, the mother treats this option as in itself problematic: the administrative task of having to alternate between dealing with the father and the SSIA “takes strength and energy.” This illustrates how the new maintenance regulations enable liable parents to shape the conditions under which resident parents receive maintenance and impose strain upon them. Descriptions like these thus show how abuse may be entangled with negligence or carelessness, making these difficult to distinguish from each other, especially when considering how the father's behavior may have negative effects on the mother's finances.

The next call illustrates another aspect of this, as the mother describes the stressful situation in explicit terms and provides the case officer with information about dysfunctions and conflict:

Ex. 6 (call 2020)

P: He won’t even talk to me. He just fights so it is impossible to agree with him. If you, like, put the decision in his hands he won’t pay a single penny. He doesn’t even talk to his son…

CO #4: But if we give him your account number and he makes the deposit to you, would that be OK?

P: But it has been like that all along

CO: Well there is this new change in the law (…) Just because you don’t think that it will work, or because you don’t want him to pay directly to you because you want it to go through the SSIA…

P: But it won’t work! He won’t pay (…) so it is not going to work. What am I going to do if he won’t even talk to me? We have no contact, nothing. How am I supposed to agree with him?

CO: Yes, but we can help you by passing on your bank account number. And we can inform him that he shall deposit [amount] straight to you

P: Yes. And he is supposed to do this every month, or..?

CO: Yes

P: Well then he’ll be like “OK” but he won’t do it.

In this call, the mother clearly depicts the father's prospective nonpayment as deliberate: “he won’t pay a single penny.” However, the case officer treats this as a practical problem rather than a possible indication of abuse, by suggesting that the SSIA can give the father the mother's bank details. In response to the mother's objection, the case officer also formulates the new regulations as coercive, leaving little possibility for the mother to impact the decision. There is no mention of the possibility of a “special reasons” exemption, and the case officer's suggestion that the mother may apply for maintenance support again (with an expected processing time of 6–8 weeks) if the father does not pay as expected does not recognize how this could negatively impact her and her son's financial situation.

The analysis in this section has illustrated the difficulties of distinguishing financial abuse from negligence or carelessness, showing how case officers treat concerns about irregular payments as a practical matter rather than a possible indication of IPV. This is perhaps not so surprising in a social insurance setting where financial problems are administered bureaucratically, with little leeway for case officers to adhere to the lifeworld problems of clients (cf., Flinkfeldt, 2020). The reformed maintenance regulations, however, presuppose that case officers will be able to make such distinctions in situated encounters as it is important when assessing the possibility of special reasons. Meanwhile, most case officers lack training or professional experience of issues relating to IPV and may not recognize the forms financial violence may take and how it may affect the children. Child abuse is not explicitly mentioned in relation to assessing special reasons in the preparatory works leading up to the reform, but based on existing knowledge, it can be assumed that forced cooperation between parents in a conflict-ridden or abusive situation may be harmful for them. Previous research has shown that children who have witnessed violence between their parents tend to avoid contact with an abusive parent, and it has therefore been suggested that limited contacts and as little parental cooperation as possible in all matters regarding custody and visitation should be prioritized issues in order to protect the child (see, e.g., Eriksson and Näsman, 2011; James-Hanman & Holt, 2021; The Swedish Ombudsman for Children, 2005). Although issues like these are not the SSIA's main focus or area of responsibility, it could be noted that the maintenance reform may contribute, directly or indirectly, to reentering an abusive parent into a child's life. It is beyond the scope of this study to speculate further about what the reform has entailed for children in families with a history of abuse, but it is reasonable to assume that financial abuse may affect the children both emotionally and financially.

According to studies investigating the effects of the reform, the case officers had not been offered any substantial capacity building regarding IPV at the point in time when recordings were made (Fernqvist, 2020; ISF, 2019), which suggests that they were unprepared for handling these matters. A related aspect is how case officers fail to recognize financial transactions as contact, which is the topic of the next section.

### Case Officers Fail to Recognize Financial Transactions as a Mode of Parental Contact

Although the maintenance reform rests on assumptions about separated parents’ ability and will to cooperate for the best interest of their children, case officers across our data tend to stress how the private arrangements imposed by the reform do not entail *contact*. When parents raise contact as a concern, case officers treat this as a practical problem by offering to pass on bank account numbers or suggesting that the liable parent can transfer money to the resident parent's phone (via the Swedish bank transfer service Swish), treating this as contact-free cooperation (similarly to what we saw in example 6). However, we argue that these transactions in fact constitute a form of contact which could be uncomfortable or otherwise problematic for mothers with previous experiences of abuse. In addition, the seemingly mundane business of money transfers could be used to exert abuse (Natalier, 2018).

In the example below, the mother is notably upset and cries throughout the call when addressing the relationship with the children's father and the anticipated difficulties of being required to manage the maintenance arrangements themselves:

Ex. 7 (call 1834)

P: He is not well … in the head. … We won’t be able to talk to each other and he is not going to pay…

CO #1: No…

P: It's very simple (…) that's what it's been like the whole time.

CO: In fact, you will not need to have any contact with each other, because, you know, he can swish money to your account via your phone number for example

P: (cries) Who will make sure that he does this! And who will tell him, because he won’t do it.

In response to the mother's strong objections, which rule out both that the father will pay and that they will be able to have contact, the case officer responds that they will not, in fact, need to have any contact—all that is required is that the father transfers money via the mother's phone. Not only does the case officer ignore the need to first reach an agreement about the maintenance sum, but she also treats cell phone transfers as not being a form of contact. Similarly, example 8 is from a call where the mother has objected to the new scheme, displaying concern about having to deal directly with the father. Like in the previous example, the case officer suggests that the SSIA can pass on the mother's bank details to the father:

Ex. 8 (call 1765)

CO #4: If we give him your bank account number…

P: But I just want to ask you something before all this … If I never get any money from him, what do I do then?

CO: Then you send us a new application [for maintenance support]

P: Well, ok, no that feels rather troublesome. I know we have to follow certain rules but you say that you have our backs and this is not painless for everybody.

This example illustrates how giving out one's bank account number may be seen as problematic, as the mother suggests that the SSIA is not sufficiently working to protect those who need them to “have our backs,” calling into question the consequences of the reform. Similarly, the next example illustrates how mothers in our data express concern about such direct payments:

Ex. 9 (call 1988)

CO #1: Even if you feel that you have strong reasons for having the maintenance via the SSIA it is not certain that my colleagues or those who administer the case think that it is enough

P: No…What would be considered strong enough reasons for those who decide..?

CO: It is more about if there has been threats or violence in the relationship

P: OK. Yeah, no, I just get a knot in my stomach when I think about leaving my bank information to him, and he is supposed to transfer money to me every month?

CO: Yes

P: And if he won’t do it on the exact same date, I’ll have to call and text him and ask him if he has made the transfer? I just feel like, nooo…

Despite the mother's extensive account of various problems with the children's father previously in the call (some of which were displayed in example 1), the case officer raises the possibility that these will not suffice to warrant continued maintenance support via the SSIA, treating the mother's experiences as not indicative of IPV. In response, the mother describes a physical reaction to the situation—a “knot in my stomach”—caused by having to share her bank details with him. She also raises the concern that additional communication will be required to actually get the money from him, which she resists. In what follows (not included in excerpt), the case officer encourages the caller to try direct maintenance transfers with the father and instructs her to text the father:

Well, I think that you should just text your bank information to the father. Just the account number will do, and then you refer to “with reference to the decision made by the SSIA to pay directly to me, here is my account number.”

Thus, the case officer does not raise the question of possible abuse, which might have warranted a special reasons exemption, but reduces the mother's apprehension towards the arrangement as insecurities regarding payment technicalities.

In sum, the institutional response to concerns about reinstated contact is passing on bank account numbers or, as in the example above, encouraging communication via text messages, also in cases where the mother has described potentially violence-implicative matters. As shown in previous research, the maintenance reform has enabled novel arenas for financial abuse as bank transfers can be labeled in abusive ways (Fernqvist, 2020) and it has also been made evident that within the Swedish context, contact arrangements related to the children can become arenas for threats and other forms of abuse (Bruno, 2018; Diesen, 2016; Lundgren et al., 2001). Worry of renewed or escalated conflict and abusive acts associated with contact regarding maintenance is therefore not likely to be unfounded, and our analysis suggests that the SSIA's definition of contact, which apparently does not include direct bank payments or verbal communication via text messages, may be too narrow.

## Discussion

After the maintenance reform in 2016, talking about violence has become of the job for case officers at the SSIA in order to assess maintenance cases and determine which parents are eligible for a continued right to maintenance support. In our data, indications of possible abuse are seldom handled as such by the case officers and in no case is an account of possible financial violence treated as possible abuse. Our analysis shows that first-line case officers seldom asked follow-up questions (see also Tegler et al., 2023), which may have to do with a restricted discretionary space or lack of awareness of mechanisms and manifestations of IPV. Although parents convey, e.g., systematically delayed payments or conflicts in relation to maintenance disagreements, they typically did not themselves define such experiences as abuse. This could probably be linked to a general lack of awareness of what financial abuse is and the forms it may take. Since experiences defined as IPV (such as financially controlling behavior and/or financial abuse) could entail that the resident parent gets to keep the maintenance transfers via the SSIA and avoid increased financial vulnerability, parental capacity building in this area is important. In addition, it is crucial that all case officers have the ability and the mandate to explore if behaviors portrayed as financial negligence may be an indication of abuse.

It should also be noted that during the years following our data collection, the SSIA has developed guidelines for how to facilitate disclosures of violence in all forms. This suggests that the case officers may now be less likely to accept accounts of, e.g., financial negligence at face value than they were at the time of the reform when our data were recorded. However, the present guidelines do not address how vague or implicit accounts in general should be dealt with and, as shown in our data, accounts that could be indicative of financial abuse are almost always imprecise. It is therefore not certain that these particular issues are handled better after the guidelines were implemented. Given the scope of the study, it has not been possible to determine whether or not accounts of negligence in relation to maintenance payments are in fact expressions of abuse, since our analysis relies solely on the recorded calls. This could be viewed as a limitation of this study. However, accounts that could indicate abuse are very rarely handled as such, which calls for further research and capacity building within Swedish social insurance. Following Hakovirta and Skinner (2021), we argue that analyzing welfare state institutions may provide useful insights on how norms regarding parental responsibilities postseparation, and particularly negotiations about cooperation, are established and applied.

Even though the new legislation takes into account experiences of IPV via the special reasons exemption, we have argued elsewhere that this part of the policy is not practically realized on the street level, as case officers fail to pick up possible abuse or actively discourage such tellings (Tegler et al., 2023). It is also likely that increased contact and cooperation postseparation may reactualize abuse, both in terms of an actual risk of reoccurring abusive events and in the form of fear, worry, or discomfort in relation to renewed contact (or a risk thereof). This is not least relevant in the context of financial issues and how they may induce conflict postseparation, in spite of attempts in welfare state institutions to prevent it (see also Natalier, 2018).

Similarly to a previous study by Eriksson and Ulmestig (2021), our study demonstrates how financial abuse is visible in Swedish welfare state operations, which suggests that practitioners and policymakers need to get a better understanding of this form of abuse and how it is linked to other forms of IPV. Our analysis has shown that case officers often encouraged parental contact or framed money transactions and text messages as something *other* than contact, thus treating payment problems as a strictly practical matter rather than something possibly linked to abuse. Meanwhile, previous studies have shown that maintenance payments can be used as a platform for verbal abuse and that financial violence has become part of maintenance support cases (Fernqvist, 2020; ISF, 2019), which is also evident in our data. Following Natalier (2018), it could be argued that the Swedish maintenance reform has entailed a form of welfare state facilitated—structural—abuse, since the parents in our data did not have to be in contact with each other whatsoever prior to the reform.

The reform—and presumptions regarding parental cooperation that constituted its foundation—initially generated concerns about the wellbeing of mothers and children with experiences of IPV. The risks associated with the reform should, therefore, not have come as a surprise for the SSIA, and it could be expected that case officers who received an increased volume of calls in the early stages of the reform, often from irritated, worried, and scared parents, were prepared for this to some extent. As shown in previous research, abuse exerted through administrative or legal processes postseparation may have a severe impact on mothers and children (Cook, 2021; Elizabeth, 2017; Miller & Smolter, 2011; Natalier, 2018). Despite good intentions regarding children’s best interests and normative assumptions about parental cooperation as starting point, our findings suggest that the Swedish maintenance reform has had palpable outcomes for some resident parents due to possible abuse in relation to the handling of finances. The reform has made it crucial to distinguish violence from other forms of conflict, or negligence, but it is clear that subtle expressions of abuse, which is often the case with financial violence, are very seldom framed as such in maintenance calls. Making financial abuse more visible as a form of structural violence in legislation, policy, and practice is therefore important, both for research and work executed in welfare state institutions.
